# Boosting the detection performance of severe acute respiratory syndrome coronavirus 2 test through a sensitive optical biosensor with new superior antibody

**DOI:** 10.1002/btm2.10410

**Published:** 2022-09-16

**Authors:** Chih‐Yen Lin, Wen‐Hung Wang, Meng‐Chi Li, Yu‐Ting Lin, Zih‐Syuan Yang, Aspiro Nayim Urbina, Wanchai Assavalapsakul, Arunee Thitithanyanont, Kai‐Ren Chen, Chien‐Cheng Kuo, Yu‐Xen Lin, Hui‐Hua Hsiao, Kun‐Der Lin, Shang‐Yi Lin, Yen‐Hsu Chen, Ming‐Lung Yu, Li‐Chen Su, Sheng‐Fan Wang

**Affiliations:** ^1^ Department of Medical Laboratory Science and Biotechnology Kaohsiung Medical University Kaohsiung Taiwan; ^2^ Center for Tropical Medicine and Infectious Disease Research Kaohsiung Medical University Kaohsiung Taiwan; ^3^ School of Medicine, College of Medicine National Sun Yat‐Sen University Kaohsiung Taiwan; ^4^ Division of Infection Disease, Department of Internal Medicine Kaohsiung Medical University Hospital Kaohsiung Taiwan; ^5^ Thin Film Technology Center National Central University Taoyuan Taiwan; ^6^ Optical Sciences Center National Central University Taoyuan Taiwan; ^7^ Department of Microbiology, Faculty of Science Chulalongkorn University Bangkok Thailand; ^8^ Department of Microbiology, Faculty of Science Mahidol University Bangkok Thailand; ^9^ Department of Optics and Photonics National Central University Taoyuan Taiwan; ^10^ TeraOptics Corporation Taoyuan Taiwan; ^11^ Division of Hematology and Oncology, Department of Internal Medicine Kaohsiung Medical University Hospital Kaohsiung Taiwan; ^12^ Division of Endocrinology and Metabolism Kaohsiung Medical University Hospital, Kaohsiung Medical University Kaohsiung Taiwan; ^13^ Department of Laboratory Medicine Kaohsiung Medical University Hospital Kaohsiung Taiwan; ^14^ Hepatobiliary Section, Department of Internal Medicine, and Hepatitis Center Kaohsiung Medical University Hospital Kaohsiung Taiwan; ^15^ General Education Center Ming Chi University of Technology New Taipei City Taiwan; ^16^ Organic Electronics Research Center Ming Chi University of Technology New Taipei City Taiwan; ^17^ Department of Medical Research Kaohsiung Medical University Hospital Kaohsiung Taiwan

**Keywords:** monoclonal antibody, PS‐SPR, SARS‐CoV‐2, spike, spike rapid antigen test, target‐captured ELISA

## Abstract

The severe acute respiratory syndrome coronavirus 2 (SARS‐CoV‐2) virus emerged in late 2019 leading to the COVID‐19 disease pandemic that triggered socioeconomic turmoil worldwide. A precise, prompt, and affordable diagnostic assay is essential for the detection of SARS‐CoV‐2 as well as its variants. Antibody against SARS‐CoV‐2 spike (S) protein was reported as a suitable strategy for therapy and diagnosis of COVID‐19. We, therefore, developed a quick and precise phase‐sensitive surface plasmon resonance (PS‐SPR) biosensor integrated with a novel generated anti‐S monoclonal antibody (S‐mAb). Our results indicated that the newly generated S‐mAb could detect the original SARS‐CoV‐2 strain along with its variants. In addition, a SARS‐CoV‐2 pseudovirus, which could be processed in BSL‐2 facility was generated for evaluation of sensitivity and specificity of the assays including PS‐SPR, homemade target‐captured ELISA, spike rapid antigen test (SRAT), and quantitative reverse transcription polymerase chain reaction (qRT‐PCR). Experimentally, PS‐SPR exerted high sensitivity to detect SARS‐CoV‐2 pseudovirus at 589 copies/ml, with 7‐fold and 70‐fold increase in sensitivity when compared with the two conventional immunoassays, including homemade target‐captured ELISA (4 × 10^3^ copies/ml) and SRAT (4 × 10^4^ copies/ml), using the identical antibody. Moreover, the PS‐SPR was applied in the measurement of mimic clinical samples containing the SARS‐CoV‐2 pseudovirus mixed with nasal mucosa. The detection limit of PS‐SPR is calculated to be 1725 copies/ml, which has higher accuracy than homemade target‐captured ELISA (4 × 10^4^ copies/ml) and SRAT (4 × 10^5^ copies/ml) and is comparable with qRT‐PCR (1250 copies/ml). Finally, the ability of PS‐SPR to detect SARS‐CoV‐2 in real clinical specimens was further demonstrated, and the assay time was less than 10 min. Taken together, our results indicate that this novel S‐mAb integrated into PS‐SPR biosensor demonstrates high sensitivity and is time‐saving in SARS‐CoV‐2 virus detection. This study suggests that incorporation of a high specific recognizer in SPR biosensor is an alternative strategy that could be applied in developing other emerging or re‐emerging pathogenic detection platforms.

## 1 INTRODUCTION

The acute respiratory disease, coronavirus disease 2019 (COVID‐19) was initially reported in December 2019 in China.^1^, ^2^, ^3^ The etiological agent was then identified and named the Novel Beta Coronavirus (severe acute respiratory syndrome coronavirus 2 [SARS‐CoV‐2]).^2^, ^4^ It is the seventh known coronavirus to infect humans; four of these coronaviruses (229E, NL63, OC43, and HKU1) reportedly only cause slight symptoms of the common cold.^5^, ^6^, ^7^ Conversely, the other three, SARS‐CoV, MERS‐CoV, and SARS‐CoV‐2, are able to cause severe symptoms and even death.^5^, ^8^, ^9^ After more than 100 million people were affected by this novel disease, on March 11, 2020 it was declared a global pandemic by WHO.^10^ SARS‐CoV‐2 has a single‐positive strand RNA genome (~29.8 kb) encoding four structural proteins: spike (S), envelope (E), matrix (M), and nucleocapsid (N).^11^, ^12^ The entry of SARS‐CoV‐2 into host cells is through an interaction between the receptor‐binding domain (RBD) of S1 subunit of spike protein and the peptidase domain of angiotensin‐converting enzyme 2 (ACE2).^13^, ^14^ To date, several SARS‐CoV‐2 variants have been detected and some variations have been proven to strengthen the interaction between SARS‐CoV‐2 RBD and ACE2.^15^, ^16^, ^17^, ^18^ From clinical observation, COVID‐19 patients present clinical manifestations ranging from asymptomatic, common‐cold symptoms to SARS that requiring immediate medical intervention.^19^, ^20^, ^21^ Therefore, the prompt and early diagnosis of SARS‐CoV‐2 infection is critical to control disease progression and dissemination.

The diagnosis of COVID‐19 is based on the clinical and epidemiological history of the patient and ancillary examination findings. Currently, there are several assays developing for COVID‐19 diagnosis including the conventional virus culture, serological assays and molecular‐based diagnoses.^22^, ^23^ Since SARS‐CoV‐2 has a considerable mortality rate (0.13–6.22%) and classification in Risk Group 3 organism by WHO and U.S. CDC, virus culture and isolation were previously viewed as gold standard assay requested to be conducted in biosafety level 3 (BSL‐3) laboratories.^24^, ^25^ However, this facility requirement limits SARS‐CoV‐2 virus detection and diagnosis using conventional virological assays. To conveniently study the properties and characteristics of SARS‐CoV‐2, the SARS‐CoV‐2 pseudovirus system, which exerted similar envelope function as SARS‐CoV‐2 real virus and could be handled in BSL‐2 laboratory was developed.^26^ In addition, the pseudoviruses also become a useful and safe virological tool for developing SARS‐CoV‐2 diagnostic assay.

Regarding COVID‐19 diagnosis, WHO and U.S. CDC suggested that the viral RNA detection by real‐time reverse transcription polymerase chain reaction (qRT‐PCR) is considered as gold standard for SARS‐CoV‐2 early diagnosis.^27^ Despite viral RNA detection using qRT‐PCR has high sensitivity, false‐negative qRT‐PCR results still arise due to improper collection of clinical specimens or poor handing of specimens during testing.^22^, ^28^ In addition, qRT‐PCR requires operation in hospital laboratories along with a rigorous stepwise process, highly trained technicians and is time‐consuming.^29^, ^30^, ^31^ Commercial COVID‐19 rapid test was successfully introduced as an alternative to qRT‐PCR, for the qualitative detection of SARS‐CoV‐2 nucleocapsid protein (N protein) based on lateral flow immunoassay. Although the costs and assay time dropped remarkably, poor sensitivity and low specificity remain as their limitations.^32^, ^33^, ^34^ In order to identify early infections effectively, and control the spread of diseases, a rapid and simple biosensing platform with high sensitivity and specificity for diagnosed infections with SARS‐CoV‐2 is urgently required.

Surface plasmon resonance (SPR) biosensors are considered as a promising alternative for highly sensitive detection of SARS‐CoV‐2, which belong to a kind of PCR‐free detection method and have been applied to detect clinically relevant viruses sensitively and rapidly.^35^, ^36^, ^37^ SPR technique works by measuring the change in the refractive index adjacent to the metallic sensing surface as a result of binding of analyte. Thus, SPR biosensors allow real‐time and label‐free detection of biomolecular interactions between the analyte and the immobilized bioreceptor (e.g., antibodies, aptamer, and nucleic acids).^38^, ^39^ In addition, it is suitable for detecting macromolecules like viral or bacterial pathogens which contribute to a significant binding‐induced refractive index change.^40^, ^41^, ^42^, ^43^ Moreover, among the four practical SPR sensing approaches of intensity, wavelength, angle, and phase, the phase‐sensitive (PS) SPR generally provide higher sensitivity since the phase change is more acute than intensity change when SPR phenomenon occurs.^44^, ^45^, ^46^, ^47^ Nevertheless, the PS‐SPR usually requires complex optical configurations or specific techniques for measuring phase due to the high optical frequencies.^45^, ^46^, ^47^

In view of this, we previously developed a simple and effective strategy for measuring the SPR phase shift based on simultaneous polarization measurement. The phase information can be extracted by a camera just in a single snapshot with no need of complicated optical alignment. The proposed PS‐SPR has been successfully implemented to the detection of glyphosate, with low molecular weight and the most frequently used herbicide worldwide, for a detection limit of 15 ng/ml (0.015 ppm).^47^ Although, the PS‐SPR offers good performance in detecting small molecules of glyphosate, with the detection limit below the worldwide strictest residual level, the value is not outstanding enough to detect other proteins. It may be limited by the affinity of the capture receptors, which is known as a significant issue that affects the assay performance for affinity‐based biosensing systems.^48^, ^49^ Therefore, in order to improve the sensitivity and specificity of the proposed PS‐SPR biosensor on SARS‐CoV‐2 detection, we generate a well‐characterized novel monoclonal antibody (mAb) via conventional hybridoma technique. This sensing platform not only exhibit excellent sensitivity and selectivity toward the SARS‐CoV‐2 S‐protein and pseudovirus, but also offers a simple and rapid detection of clinical specimens within 10 min. We believe that this sensitive and rapid detection approach has the potential to be applied for clinical COVID‐19 diagnosis.

## 2 EXPERIMENTAL SECTION

### 2.1 Ethics statement

The study was approved by the Institutional Ethics Committee of the Kaohsiung Medical University (KMUHIRB‐G(I)‐20190048). All procedures were conducted according to committee regulations. Regarding mAb generation, we followed procedures according to the Institutional Animal Care and Use Committee (IACUC) guidelines. The protocols of animal use and handle were estimated and approved by the Institutional Animal Care and Use Committee of Kaohsiung Medical University. When immunized mice showed the highest titers of anti‐sera against spike proteins, they were considered to have reached the end point of this experiment. The mice were euthanized humanely to ameliorate suffering, following the IACUC guideline (No.109062).

### 2.2 SARS‐CoV‐2 spike‐ACE2 protein–protein interaction assay, generation of SARS‐CoV‐2 pseudovirus and viral infectivity assay

The SARS‐CoV‐2 spike‐ACE2 protein–protein interaction assay was described elsewhere.^50^ SARS‐CoV‐2 pseudovirus and infectivity assay was generated according to the protocol published previously.^51^ The details of these assays were described in the Supplementary data.

### 2.3 Anti‐S monoclonal and polyclonal antibody preparation

The recombinant SARS‐CoV‐2 spike S1 protein (Wuhan‐Hu‐1) (Genbank Accession No. YP_009724390) generated from HEK293T cell expressing system were obtained from the vender (SinoBiological, Cat. No. 40591‐V08H). The recombinant S1 proteins were used to generate monoclonal antibodies (mAb). Briefly, the BALB/c mice were immunized intraperitoneally with 2.5 μg of recombinant S1 proteins in 0.25 ml of PBS, which was emulsified with an equal volume of complete Freund's adjuvant (Sigma Aldrich, Cat. No. F5881). Twice intraperitoneal booster of the same dose of recombinant S1 proteins emulsified with incomplete Freund's adjuvant (Sigma Aldrich, Cat. No. F5506) was performed. A final boost was given 3 days before splenocytes were collected and fused to NS‐1 myeloma cells, as described previously.^52^, ^53^ Hybridomas producing antibodies were screened by serial dilution and several clones that produced specific individual antibodies were selected.^52^, ^53^

Regarding the generation of S polyclonal antibodies, the New Zealand white rabbits were immunized and boosted (three or four times total) with 50 μg recombinant SARS‐CoV‐2 S1 proteins in 0.25 ml PBS emulsified with an equal volume of complete/incomplete Freund's adjuvant. After confirming increases in antibody titers against recombinant SARS‐CoV‐2 S1 protein large amounts of rabbit blood were drawn for assays.

### 2.4 Specific epitopes in SARS‐CoV‐2 S‐protein recognized by spike mAbs

The epitopes recognized by selected S‐mAbs were determined. Phage display for epitope mapping was described previously. A phage‐displayed 7‐mer peptide library was purchased from New England Biolabs (NEB), Inc. (Cat. No. E8102L). Phage display bio‐panning procedures were performed according to the NEB manufacture's protocol.

### 2.5 SPR chip preparation

In this study, a carboxymethyl‐dextran CM5 chip was used as the SPR chip and purchased from Cytiva (Marlborough, MA). The modification of the SPR chip surfaces is executed using the amine coupling procedure. In brief, the carboxyl groups on the chip surface were first activated with 1‐ethyl‐3‐(3‐dimethylaminopropyl)‐carbodiimide hydrochloride and *N*‐hydroxysuccinimide for about 10 min. Then, these groups covalently coupled to the capture antibody, S‐mAb, at a concentration of 30 μg/ml for about 30 min. Finally, the remaining active carboxyl groups were deactivated using a blocking agent (ethanolamine‐HCl, ETH) for about 7 min to prevent further coupling reaction. The amine coupling kit was obtained from Cytiva as well.

### 2.6 Measurement using the PS‐SPR biosensor

**FIGURE 1:**
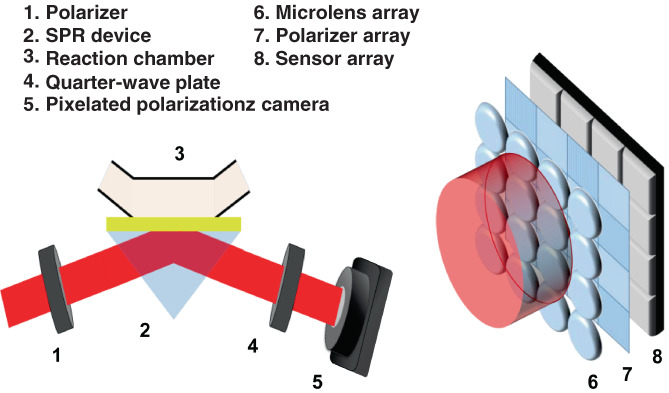
Schematic illustration of the phase‐sensitive surface plasmon resonance (SPR) based on simultaneous polarization measurement with common‐path interferometry. The system equips a polarizer, a Kretschmann‐based SPR device, a quarter‐wave plate and a pixelated polarization camera which is comprised of a polarizer array, a microlens array, and a sensor array (right panel)

The optical setup of the PS‐SPR biosensor consists of a linearly polarized He‐Ne laser with wavelength of 633 nm, a polarizer for tuning light polarization, a Kretschmann‐based SPR device, and a detection system comprised of a quarter‐wave plate (QWP) and a pixelated polarization camera (PPC) (see Figure 1). The detection principle of the PS‐SPR is based on phase‐shift algorithms. Briefly, the reflected light undergoing SPR was converted into two orthogonal circular polarizations by the QWP, and then occurred optical interference on the PPC with a polarizer array and a microlens array. The PPC can simultaneously provide the intensity signals of four polarization directions, 0, 45, 90, and 135° such that the phase difference can be effortlessly acquired in a single snapshot. The principle is explained in detail in our previous study.^47^ All analytes flowed through the prepared SPR chip to interact with the immobilized S‐mAb at a constant flow rate of 40 μl/min. The testing sample including spike protein, SASR‐CoV‐2 pseudovirus and clinical COVID‐19 nasopharyngeal swab.

### 2.7 Establishment of the homemade target‐captured ELISA

The S‐mAb and S‐pAb (spike‐polyclonal antibody) were used to establish a target‐captured ELISA assay. The detailed protocol of the target‐captured ELISA was described previously.^54^ Briefly, 96‐well round‐bottom microtiter plates (Nunc, Roskilde, Demark) were coated with 1 μg purified S‐pAb in 100 μl carbonate buffer (73 mM sodium bicarbonate and 30 mM sodium carbonate) and incubated at 37°C for 1 h or at 4°C overnight. The plates were washed three times with PBST and blocked with PBST that contained 5% bovine serum albumin at 37°C for 1 h. After washing three times with PBST, the S‐pAbs coated 96‐well plates could be used for the SARS‐CoV‐2 spike protein or pseudovirus detection. The standard S1 proteins and qRT‐PCR quantified SARS‐CoV‐2 pseudovirus were both used as the detection target for the target‐captured ELISA. The diluted and quantified SARS‐CoV‐2 pseudovirus in PBS or mimic samples (the SARS‐CoV‐2 negative throat swab samples) were added into 96‐well plates and then incubated at 37°C for 2 h. After washing five times with PBST, the 100 μl of 1000‐fold dilution S‐mAb (1 mg/ml) were added to the wells and incubated for 1 h at 37°C. After five washes with PBST, 100 μl HRP‐conjugated goat anti‐mouse IgG antibody (Sigma‐Aldrich, Cat. No. 665739) was added into each well at 37°C for 1 h. After washing five times with PBST, color development was performed by the addition of 100 μl of freshly prepared TMB solution (ThermoFisher); the absorbance at 450 nm was read with an ELISA reader (Tecan, Switzerland). Furthermore, the homemade target‐captured ELISA was compared the detection sensitivity with commercial SARS‐CoV‐2 Spike Protein S1 ELISA Kit (Cell Biolabs Cat. No.VPK‐5155). The steps and result interpretation were carried out according to the manufacturer's protocol (Cell Biolabs Cat. No.VPK‐5155).

### 2.8 Measurement of the SARS‐CoV‐2 pseudovirus using spike rapid antigen test

SARS‐CoV‐2 spike rapid antigen test (SRAT) was performed using our generated mAb and pAb against S proteins. Preparation of SRAT strip was entrusted by BIOTOOLS company. SRAT strips consisted of a sample pad, a conjugate pad, a nitrocellulose membrane and an absorbent pad. The conjugate pad contained the S‐mAb‐conjugated red cellulose nanobeads whereas the test line and control line contain S‐pAbs and anti‐IgG Abs, respectively. The testing procedure was conducted according to manufacturer's protocol. Briefly, the SARS‐CoV‐2 S1 protein or pseudovirus was diluted in PBS or mimic samples (the SARS‐CoV‐2 negative throat swab samples). The analytes were mixed with extraction buffer (to make the mixture in a total of 100 μl) and then added to the reactive strip at room temperature for 15 min. The reactive bands of the SARS‐CoV‐2 as well as the control were observed.

### 2.9 Measurement of the SARS‐CoV‐2 pseudovirus using qRT‐PCR

The qRT‐PCR was used to detect and quantify the SARS‐CoV‐2 pseudovirus. The protocol was described elsewhere and the primers and probe used were listed as below.^55^ LTR‐Forward primer: 5′‐TGCTTAAGCCTCAATAAAGCTTGCCTTGA‐3′; LTR‐Reverse primer:5′‐TCTGAGGGATCTC TAGTTACCAG‐5′ and LTR‐probe: Fam‐5′‐AAGTAGTGTGTGCCCGTCTGT‐3′‐Qsy. Briefly, infected cell lysates were collected and subjected to RNA extraction using TOOLS EsayPrep total RNA kit (Cat.No. DPT‐BD19; BIOTOOLS, Taiwan). 1.0μl of 10μM LTR forward and reverse primers, 0.5μl of the corresponding probes, 5μl of extracted RNA, 12.5 μl of 2× one step RT‐PCR buffer (TOOLS One‐Step Probe RT‐qPCR Mix, Cat.No.FPT‐BC14), 1.0 μl of 25× Enzyme Mix (TOOLS One‐Step Probe RT‐qPCR Mix, Cat.No.FPT‐BC14) and an appropriate volume of Rnase‐free distilled water (dH_2_O) were mixed in a total 25 μl reaction volume. The thermal cycles were performed on a QuantStudio 3 Real‐Time PCR System (Applied Biosystems, Foster city, CA). The real‐time PCR conditions were as follows: 30 min at 50°C for RT; 5 min at 95°C, then 40 cycles at 95°C for 15 s and 55°C for 45 s for real‐time quantitative PCR. All real‐time PCRs were performed in technical triplicates for three biological replicates. [Correction added on 22 October 2022, after first online publication: “Textual corrections has been made, replacing a sentence in this paragraph”.]

## 3 RESULTS AND DISCUSSION

### 3.1 Generation of the mAb against the SARS‐CoV‐2 spike protein

Currently, SARS‐CoV‐2 spike protein has been proved as the main protein to interact with ACE2 receptor expressed on the target cells and subsequently caused the infection.^56^, ^57^, ^58^ Spike protein is the leading therapeutic and diagnostic target for vaccine, antiviral drug and novel diagnostic platform development.^59^, ^60^ We therefore intended to generate the mAb and pAb against SARS‐CoV‐2 spike protein, mainly focused on S1 domain since it contains the RBD domain and has high specificity compared to S2 subunit which comprises fusion peptide, transmembrane domain, and cytoplasm domain that are not easily detected by antibodies in natural condition.

**FIGURE 2:**
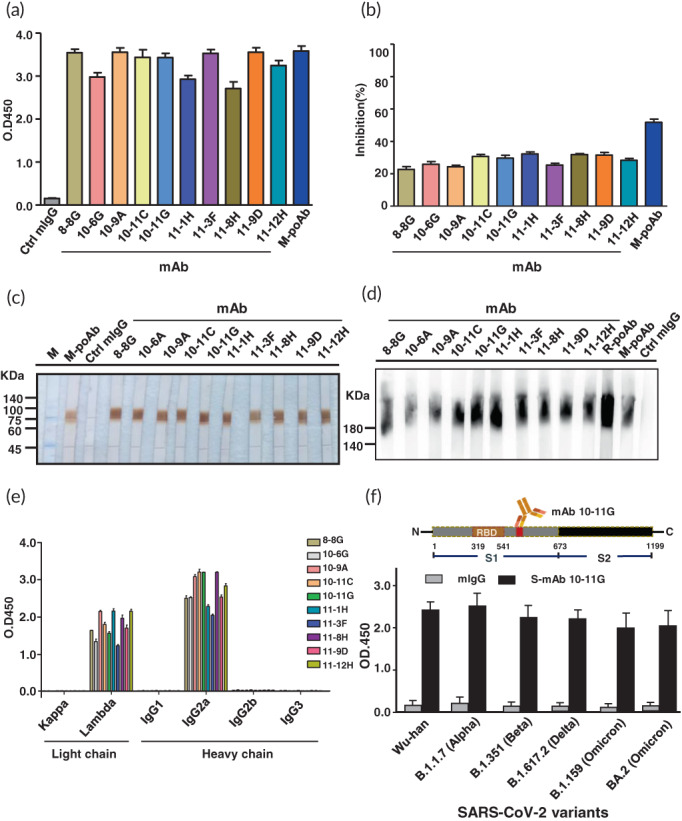
Generation and characterization of monoclonal antibodies against severe acute respiratory syndrome coronavirus 2 (SARS‐CoV‐2) spike. A series of SARS‐CoV‐2 spike monoclonal antibodies (S‐mAbs) were generated via hybridoma technique and serial dilution assay. (a) 10 S‐mAb with higher binding abilities to spike S1 protein suing ELISA were selected. (b) The viral neutralization capabilities of these 10 S‐mAbs were evaluated using spike‐ACE2 protein–protein interaction ELISA assay. The denature (c) and native (d) PAGE and immunoblotting using spike S1 protein detected by 10 selected S‐Abs are shown. (e) The isotype determination of these 10 S‐mAbs is illustrated. (f) The selected S‐mAb 10‐11G was subjected to epitope mapping with phage display assay and mapped epitope region are shown on the scheme (upper). The spike S1 protein from SARS‐CoV‐2 original strain (Wuhan strain) and its variants including B.1.17, B.1.351, B.1.617.2, and Omicron strain (including original B.1.159 and BA.2) were subjected to ELISA and detected by isotype IgG control and S‐mAb 10‐11G. The data are presented as the means ± standard deviations (SDs) of three independent biological replicates

The SARS‐CoV‐2 S1 proteins were generated from HEK293T expressing system and their protein size, immunogenicity and function were confirmed via Coomassie blue staining, immunoblotting and Spike‐ACE2 protein–protein interaction ELISA‐based assay (Figure S1). The SARS‐CoV‐2 S1 proteins were used to immunize BALB/c mice and New Zealand white rabbits to generate S‐mAbs and S‐pAbs. The production of S‐mAb was according to conventional hybridoma technique combined with serial dilution. A total of 10 S‐mAbs were selected with these S‐mAbs displaying higher binding capabilities and partial neutralization abilities (Figure 2a,b). In addition, these S‐mAbs could recognize denatured and native forms of SARS‐CoV‐2 spike protein (Figure 2c,d). Isotyping results indicated that all the S‐mAbs belonged to IgG2a and with lambda light chain (Figure 2e). The binding affinity of these S‐mAbs was measured via SPR and results indicated that mAb 10‐11G, 11‐3F, 11‐8H, and 10‐6G displayed higher binding affinity to SARS‐CoV‐2 spike protein (*K*_D_ (M) = 3.17 × 10^−7^–8.68 × 10^−7^) (Table S1). We therefore selected S‐mAb 10‐11G as candidate for developing COVID‐19 diagnostic assay and platform. Notably, the recognition epitope of S‐mAb 10‐11G was identified using phage display assay and results revealed that the epitope is located on the C‐terminal domain of S1 protein and this epitope is beside the C‐terminal region of RBD (Figure 2f). We identified that the epitope recognized by S‐mAb 10‐11G is highly conserved among past and current circulating SARS‐CoV‐2 variants (100% identity) (Figure 2f). Data from ELISA using different SARS‐CoV‐2 variant S1 protein detected by our generated S‐mAb 10‐11G found that all the previous and current predominant SARS‐CoV‐2 mutant strains, including Wuhan strain, B.1.1.7 (Alpha strain), B.1.351 (Beta strain), B.1.617.2 (Delta strain), and Omicron strains (including original B.1.159 and BA.2) could be detected by S‐mAb 10‐11G (Figure 2f). Accordingly, we suggest that this epitope recognized by our generated S‐mAb is of low mutation prone since it does not directly face the humoral antibodies or is recognized by CD8 cytotoxic T cells. Several reports have indicated that most SARS‐CoV‐2 variants have the amino acid substitutions mainly on RBD and partially occurred on the rest regions to escape the immune attack as well as increase their interacting abilities with ACE2 receptors. Although there are many studies that generate S‐mAbs and most of these studies report that their S‐mAbs targets the RBD domain with prefect viral neutralization capabilities, these S‐mAbs show low protective abilities when SARS‐CoV‐2 has mutations, especially the mutations are located on the RBD. Compared to our generated S‐mAb 10‐11G, which recognizes a conserved epitope which is located on the C‐terminal of the RBD and shows partial neutralization abilities, suggesting that this mAb is worthy for applying to COVID‐19 diagnosis and has potential for COVID‐19 therapy. To validate the high and board binding abilities of our selected S‐mAb 10‐11G, two commercial S‐mAbs were used to be compared using ELISA and immunoblotting assays. Results indicated that our generated mAb 10‐11G could recognize most SARS‐CoV‐2 spike variant proteins, where the commercial S‐mAbs only could detect certain spike variants (Figure S2). Similarly, data from immunoblotting revealed that our generated mAb 10‐11G could detect most spike variant proteins compared to commercial S‐mAbs which only detected the spike proteins from certain SARS‐CoV‐2 variants (Figure S3).

In addition, the benefits of using S‐mAb for SARS‐CoV‐2 detection and diagnosis is that spike protein has lower similarity among human coronaviruses compared to nucleocapsid protein, the current SARS‐CoV‐2 serological diagnostic target, which higher similarity among human coronaviruses. As it has been previously reported in literature, SARS‐CoV‐2 S protein shows around 76% protein identify with SARS‐CoV‐1 whereas SARS‐CoV‐2 N protein shows 90% protein identify with SARS‐CoV‐1.^61^, ^62^ These studies might hint that S protein and its corresponding antibody displays higher specificity to SARS‐CoV‐2. Indeed, our results from ELISA and immunoblotting using SARS‐CoV‐1 and SARS‐CoV‐2 indicated that our S‐mAb 10‐11G could differentiate SARS‐CoV‐1 and SARS‐CoV‐2, suggesting that our S‐mAb 10‐11G recognized a unique SARS‐CoV‐2 epitope (Figure S4).

### 3.2 Preparation SARS‐CoV‐2 pseudovirus

As mentioned before, SARS‐CoV‐2 virus is currently classified in Risk Group 3 organism and needs to be handled in BSL‐3 facility. Pseudotyped viruses that lack certain gene sequences of the virulent virus are safer and can be investigated in BSL‐2 laboratories, providing a useful virological tool for the study of SARS‐CoV‐2.^63^ SARS‐CoV‐2 pseudovirus system has been reported in many recent studies as a tool implemented into SARS‐CoV‐2 research and the development of vaccines and therapeutics.^51^, ^63^ For packing the SARS‐CoV‐2 pseudovirus, there are three main strategies, including (1) the human immunodeficiency virus (HIV‐1)‐based lentiviral packaging system,^64^, ^65^ (2) the murine leukemia virus‐based packaging system,^66^, ^67^ and (3) the VSV packaging system.^68^ Among these systems, the HIV‐1 lentiviral packaging system is currently the most widely used SARS‐CoV‐2 pseudoviral packaging system.^63^ Generally, the HIV‐1 lentiviral packaging system could be conducted via two or three plasmids co‐transfecting into the cells to produce the pseudovirus. The two‐plasmid packaging system is the most common SARS‐CoV‐2 pseudoviral packaging system, which includes a plasmid to express the SARS‐CoV‐2 S protein and another HIV‐1 backbone plasmid that expresses the packaging proteins and signals but has the envelope gene deleted.^69^ Here, we used the two plasmid strategies to generate the SARS‐CoV‐2 pseudovirus in this study. The plasmids expressing HIV core structure which contain the luciferase gene and SARS‐CoV‐2 spike protein were transfected into HEK293T. The culture supernatants containing SARS‐CoV‐2 spike pseudotyped lentivirus were collected and subjected to measurement of their titers using qRT‐PCR. Moreover, their infectious abilities were confirmed using infectivity assay and immunofluorescent staining using HEK293T expressed human ACE2 (293T‐ACE2) cells. Viral infectivity and immunofluorescent staining results indicated that SARS‐CoV‐2 pseudovirus displayed infectivity to 293T‐ACE2 cells via detection of luciferase activity as well as observation of green fluorescence from infected cells (Figure S5a,b). To further confirm the infectious properties of SARS‐CoV‐2 pseudovirus similarities with real SARS‐CoV‐2 virus, the COVID‐19 convalescent anti‐sera were used to preincubate with SARS‐CoV‐2 pseudovirus in prior to be incubated with 293T‐ACE2 cells. Results indicated that the infected cells by SARS‐CoV‐2 pseudovirus were significantly reduced in pretreated COVID‐19 anti‐sear group compared to pretreated healthy anti‐sera control group (*p* < 0.05) (Figure S5). Our data indicated that this SARS‐CoV‐2 pseudovirus could interact with ACE2‐expressing cells to cause infection and this infectivity could be neutralized by COVID‐19 convalescent anti‐sera, suggesting that this SARS‐CoV‐2 pseudovirus displayed similar infectious capability and function as SARS‐CoV‐2 isolate. Combined, the SARS‐CoV‐2 pseudovirus is suggested as an alternative tool for studying and evaluating the diagnostic and therapeutic strategy in most research and academic organizations which lack the BSL‐3 facility.

### 3.3 PS‐SPR detection of SARS‐CoV‐2 S protein

**FIGURE 3:**
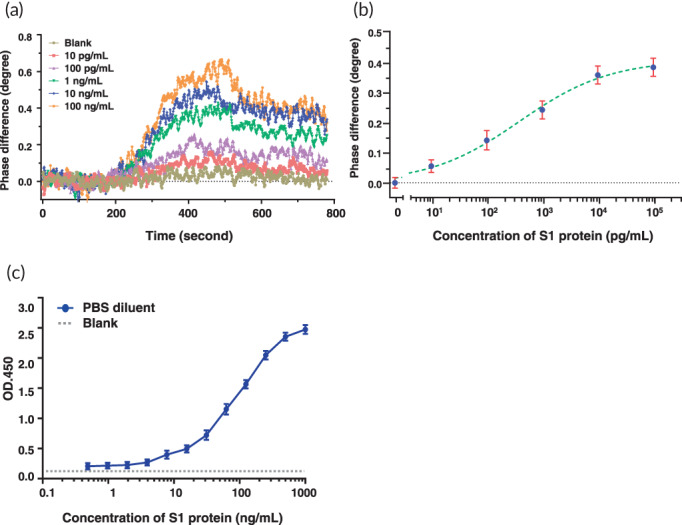
Detection limit analysis of phase‐sensitive surface plasmon resonance (PS‐SPR) and homemade target‐captured ELISA toward severe acute respiratory syndrome coronavirus 2 (SARS‐CoV‐2) spike protein. (a) The real‐time resulting SPR phase shifts for various concentration of recombinant S1 proteins. (b) The standard curve for the recombinant S1 proteins ranging from 10 pg/ml to 100 ng/ml. (c) The serial twofold dilution of recombinant S1 protein was subjected to homemade target‐captured ELISA and limit of detection concentration of S1 protein was determined. The results are presented as the means + standard deviations (SDs) of three independent biological replicates

The PS‐SPR platform was based on simultaneous polarization measurement with common‐path interferometry and a scheme of establishment is shown in Figure 1. The analytical performance of the PS‐SPR biosensor was investigated by measuring the changes in SPR phase shift with different concentrations (0.01, 0.1, 1.0, 10, and 100 ng/ml) of the recombinant S1 protein. The real‐time results of the SPR phase shifts are shown in Figure 3a, along with a growing trend with increasing analyte concentration. This behavior revealed that more targets are captured to from the ⟨S‐mAb/recombinant S1 protein⟩ complex on the SPR sensor chip. The SPR phase shift is plotted as a function of the target concentration to generate a standard curve, as displayed in Figure 3b. In this experiment, PBS solution was the dilution buffer and also treated as the control group (Mock or blank). The standard curve of the PS‐SPR biosensor is fitted using four‐parameter logistic regression, and the limit of detection of the PS‐SPR biosensor for the S1 protein was estimated to be 11 pg/ml based on the IUPAC's definition. This value is lower than that of antibody‐based ELISA (0.7 ng/ml)^70^ and that of aptamer‐based electrochemical sensor (66 pg/ml),^71^ which both assays detect the SARS‐CoV‐2 S protein. Further, when compared to the homemade target‐captured ELISA, the detection limit of the recombinant S1 protein was estimated to 0.48 ng/ml (Figures 3c and S6). Currently, spike antigen‐captured ELISAs are widely available for detection SARS‐CoV‐2 viruses. Our homemade target‐captured ELISA was used to compare the detection capabilities with commercial SARS‐CoV‐2 S1 ELISA (Cell Biolabs). Results indicated that the limit of detection of spike protein by the homemade S1 target‐captured ELISA was 0.48 ng/ml whereas the limit of detection of spike protein by commercial SARS‐CoV‐2 S1 ELISA was 31.3 ng/ml (Figure S6). These results suggested that a new mAb with high sensitivity and specificity as well as broad detection abilities is necessary for applying SARS‐CoV‐2 detection and diagnosis.

### 3.4 PS‐SPR detection of SARS‐CoV‐2 pseudovirus and clinical samples

**FIGURE 4:**
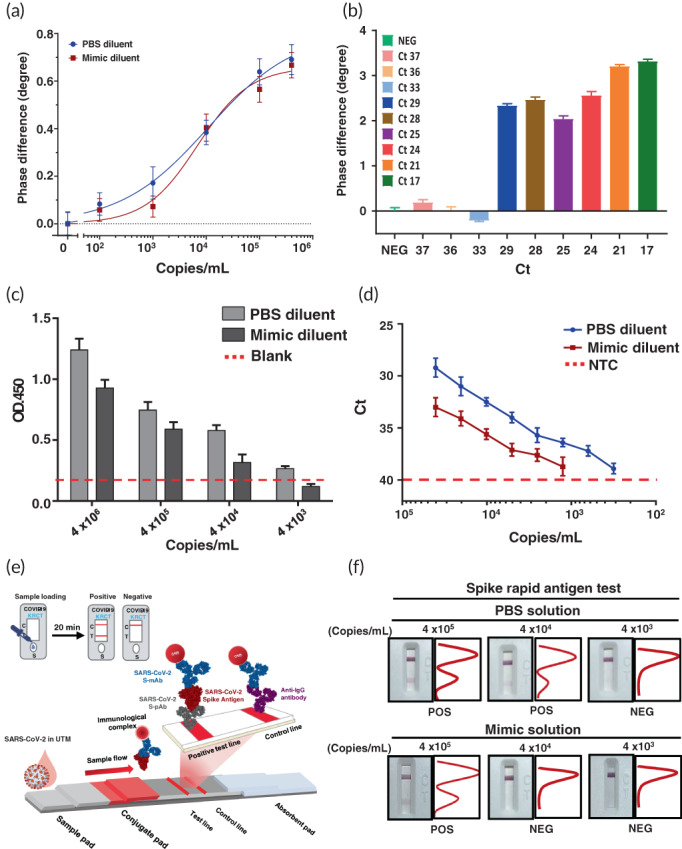
Detection limit of severe acute respiratory syndrome coronavirus 2 (SARS‐CoV‐2) using different immunoassays was validated. (a) The serial 10‐fold dilution of SARS‐CoV‐2 pseudovirus in PSB or mimic diluent was subjected to phase‐sensitive surface plasmon resonance (PS‐SPR). (b) The measured SPR phase difference caused by SARS‐CoV‐2 in the clinical samples. The serial 10‐fold and 2‐fold dilution of SARS‐CoV‐2 pseudovirus in PSB or mimic diluent was subjected to (c) homemade target‐captured ELISA and (d) quantitative reverse transcription polymerase chain reaction (qRT‐PCR) analyses, respectively. (e) The S monoclonal antibody (S‐mAb) and spike‐polyclonal antibody (S‐pAb) were used for establishment of the spike rapid antigen test (SRAT). The scheme of SRAT was consisted of a sample pad, conjugate pad, nitrocellulose membrane, and absorbent pad. The test line placed on the nitrocellulose membrane contained S‐pAb for detection of the SARS‐CoV‐2 spike protein whereas the anti‐IgG antibody was used in the control line. (f) The sensitivity analysis of spike rapid antigen test (SRAT) was conducted using SARS‐CoV‐2 pseudovirus in PSB or mimic diluent. The intensity of the test and control lines was converted to a peak histogram by an image analyzer. The representative data are shown. The results are presented as the means ± standard deviations (SDs) of three independent biological replicates

To investigate the practical feasibility of the proposed PS‐SPR biosensor for COVID‐19 detection, the experiment was performed with the SARS‐CoV‐2 pseudovirus in culture medium. The quantification of initial titers of the SARS‐CoV‐2 pseudovirus in culture supernatant was assessed by qRT‐PCR to be 4.0 × 10^6^ copies/ml. The SARS‐CoV‐2 pseudovirus stock was diluted to 4.0 × 10^5^, 1.0 × 10^5^, 1.0 × 10^4^, 1.0 × 10^3^, and 1.0 × 10^2^ copies/ml with PBS. The SPR phase shift caused from SARS‐CoV‐2 pseudovirus binding to the immobilized S‐mAb on the chip is shown in Figure S7a. It can be seen that the resulted SPR phase shift is increased as the concentration of the SARS‐CoV‐2 pseudovirus increased, and the total test time for each assay is within 15 min. The relationship between the SPR phase shift and pseudovirus concentration over the range of 1.0 × 10^2^ copies/ml to 4.0 × 10^5^ copies/ml is displayed in Figure 4a, and the result is analyzed with the four‐parameter logistic regression as well. According to the IUPAC's definition, the limit of detection of the PS‐SPR biosensor for the SARS‐CoV‐2 pseudovirus detection in PBS‐diluted culture medium was estimated to be 589 copies/ml.

Moreover, the bioapplicability of the proposed PS‐SPR biosensor to detect COVID‐19 in clinical specimens was further evaluated. In the mimic experiments, the SARS‐CoV‐2 pseudovirus stock was diluted with mimic samples (the SARS‐CoV‐2 negative throat swab samples), and the control group (Mock) was mimic samples in the absence of SARS‐CoV‐2 pseudovirus. In this experiment, the NSB reducer (10 mg/ml carboxymethyl dextran sodium salt, 0.15 M MaCl, 0.02% NaN3) was added to the samples to help reduce non‐specific binding from complex sample components onto the SPR chip surfaces since the carboxymethyl dextran in the NSB reducer has a similar structure to the dextran matrix on the sensor surface. The real‐time SPR phase shift of the interaction is shown in Figure S7b. A similar result was obtained for the standard curve of the SARS‐CoV‐2 pseudovirus detection in the mimic samples over the range of 1.0 × 10^2^ copies/ml to 4.0 × 10^5^ copies/ml, as represented in Figure 4a, and the limit of detection of the PS‐SPR biosensor for SARS‐CoV‐2 pseudovirus detection in the mimic samples was estimated to be 1725 copies/ml.

In order to demonstrate the PS‐SPR biosensor ability to be applied in clinical practice, we conducted the new experiment with the clinical COVID‐19 confirmed nasopharyngeal swabs. A total of 10 samples, including negative and Ct value ranged from 17 to 37, were carried out, and the results are shown in Figure 4b and Table S2. In Figure 4b, it is obvious to see that the SPR phase signals can be divided into two groups. One group consists of the PCR Ct value above 33 and the SPR phase signals cannot be distinguished from negative in the specimens; they are noted as “−” in Table S2. The other group consists of the PCR Ct value below 29, and the SPR phase signals are higher than the former group; they are noted as “+” in Table S2. In addition, in Figure 4b, it is only observed a weak negative relationship (*r* = −0.82) between the SPR phase signal and the PCR Ct value for the PCR Ct value below 29. We speculate on the possible reasons as follows. Restricted to regulations, the clinical specimens used in this experiment are all clinical remaining specimens discarded after exceeding the legal storage period from the hospital. Due to the different preservation conditions in each clinical specimen, the degradation of viral particles in each specimen is also inconsistent. We speculate that this is the main reason that the SPR phase signal only shows a weak negative correlation with the PCR Ct value. Meanwhile, it is also observed that the SPR phase signal in clinical samples experiment is higher than that in SARS‐CoV‐2 pseudovirus detection. It should result from the significant difference in composition of real SARS‐CoV‐2 and pseudovirus except for surface proteins such as the S1 protein. Fortunately, the proposed PS‐SPR still could be used to diagnose COVID‐19, although we cannot derive the viral concentration of each patient sample in this experiment.

### 3.5 Detection of SARS‐CoV‐2 pseudovirus using ELISA, qRT‐PCR, and rapid test assays

Next, we evaluated the detection sensitivity for the SARS‐CoV‐2 pseudovirus using the qRT‐PCR, homemade target‐captured ELISA and spike rapid test. The qRT‐PCR quantified SARS‐CoV‐2 pseudovirus (4 × 10^5^/ml) was under different dilutions using PBS or mimic samples. The results from homemade target‐captured ELISA indicated that the detection limit of SARS‐CoV‐2 pseudovirus in PBS and mimic samples were 4 × 10^3^ and 4 × 10^4^ copies/ml, respectively (Figure 4c). We also noted that homemade target‐captured ELISA exerted higher limit of detection compared with commercial S1 ELISA kit which could detect the concentration to 4 × 10^6^ copies/ml (Figure S6b,d). Additionally, the twofold serial diluted SARS‐CoV‐2 pseudovirus was subjected to qRT‐PCR analysis and results indicated that the detection limit of SARS‐CoV‐2 pseudovirus in PBS and mimic samples were 312 and 1250 copies/ml, respectively (Figure 4d).

Current SARS‐CoV‐2 antigen rapid test mainly targets the SARS‐CoV‐2 nucleocapsid (N) protein. Although N protein is abundant in SARS‐CoV‐2 virus particles, higher percentage of protein identity of SARS‐CoV‐2 N protein compared with other human coronavirus has been reported,^61^ which would lead to the necessity of double confirmation of the rapid test results with qRT‐PCR. We therefore generated the SARS‐CoV‐2 SRAT using our S‐mAb and S‐pAb (Figure 4e). The serial diluted SARS‐CoV‐2 pseudovirus was measured by SRAT and results indicated that detection limit of SARS‐CoV‐2 pseudovirus in PBS and mimic samples were 4 × 10^4^ and 4 × 10^5^, respectively (Figure 4f).

### 3.6 Evaluation of the specificity for SARS‐CoV‐2 pseudovirus detection

**FIGURE 5:**
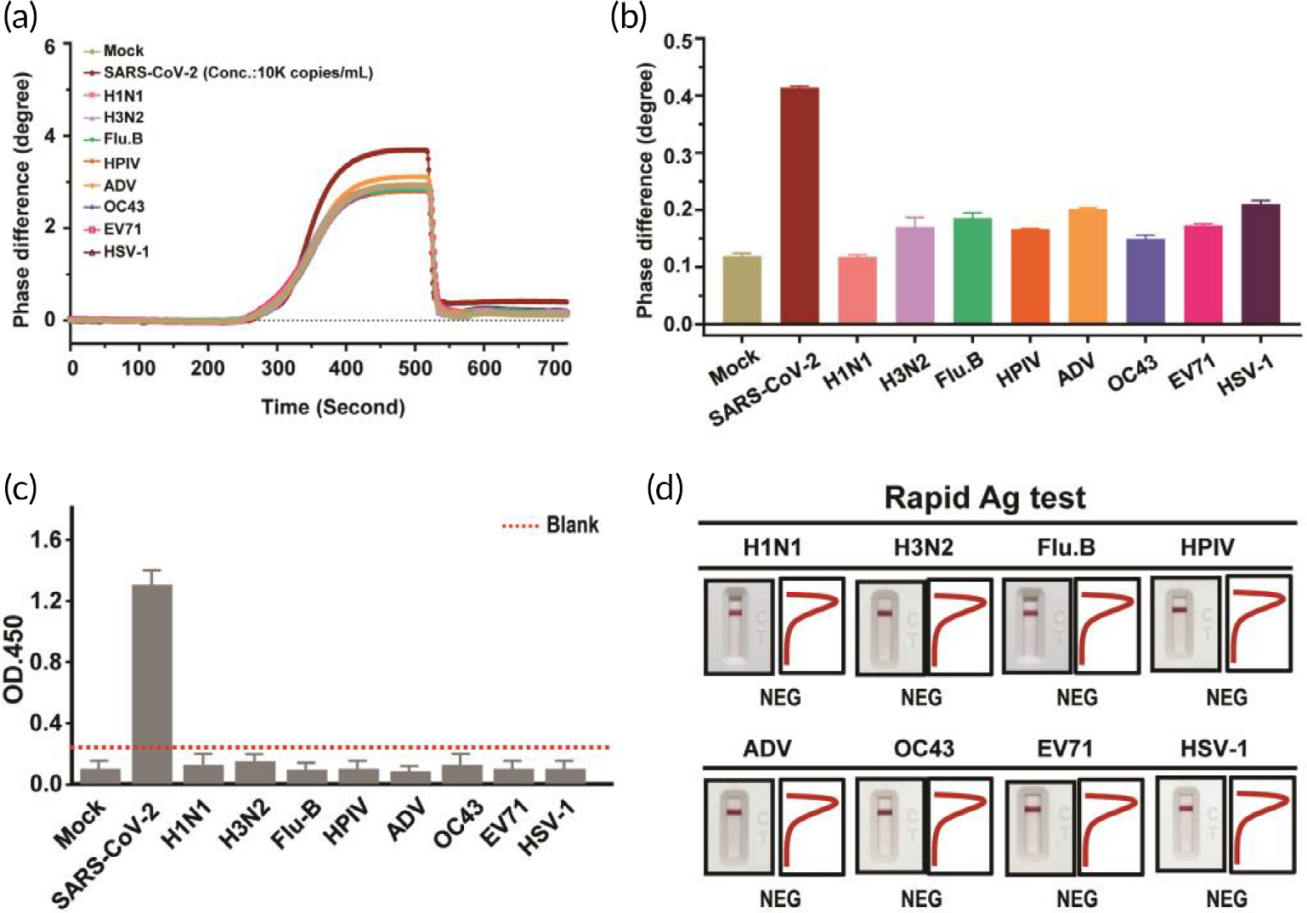
Specificity analysis of phase‐sensitive surface plasmon resonance (PS‐SPR) and homemade target‐captured ELISA and spike rapid antigen test (SRAT). Severe acute respiratory syndrome coronavirus 2 (SARS‐CoV‐2) pseudovirus and other human respiratory viruses, including influenza A H1N1 and H3N2, influenza B (Flu B), human parainfluenza virus (HPIV), adenovirus (ADV), human coronavirus OC‐43 strain (HCoV‐OC43), enterovirus 71 (EV‐71), and herpes simplex virus type‐1 (HSV‐1) were subjected to PS‐SPR (a,b), homemade target capture ELISA (c), and SRAT (d) analyses. The representative data are shown. The results are presented as the means ± standard deviations (SDs) of three independent biological replicates

The specificity of the newly generated antibody toward SARS‐CoV‐2 S protein was evaluated by the PS‐SPR biosensor and compared with homemade target‐captured ELISA and SRAT. The work was conducted with eight respiratory viruses, including influenza A H1N1 and H3N2, influenza B (Flu B), human parainfluenza virus, adenovirus, human coronavirus OC‐43 strain (HCoV‐OC43), enterovirus 71 (EV‐71), and herpes simplex virus type‐1 (HSV‐1). Experimentally, the concentrations of the eight testing viruses and SARS‐CoV‐2 pseudovirus were 1.0 × 10^5^ copies/ml and 1.0 × 10^4^ copies/ml in PBS‐diluted culture medium, respectively. The response of the PS‐SPR biosensor toward the blank test (PBS‐diluted culture medium), the eight testing viruses, and the SARS‐CoV‐2 pseudovirus are displayed (Figure 5a,b). It can be noted that the eight testing viruses reveal similar response to that of the blank, but significantly lower than that of SARS‐CoV‐2 pseudovirus. It is worth nothing if it cannot differentiated between HCoV‐OC43 and SARS‐CoV‐2, of which the clinical presentations resemble a lot to each other. Similar results were found in homemade target‐captured ELISA and SRAT, indicating that all the eight respiratory viruses were not detected by these two assays (Figure 5c,d). Taken together, these results demonstrate that the newly generated S‐mAb indeed has a high specificity in detecting the SARS‐CoV‐2 and its applicated diagnostic platform and assay also displayed high specificity.

### 3.7 Performance comparison of the PS‐SPR biosensor with ELISA, SRAT, and qRT‐PCR for SARS‐CoV‐2 pseudovirus detection

The diagnostic performance of the PS‐SPR biosensor was compared with homemade target‐captured ELISA, SRAT, and qRT‐PCR. The results demonstrated that PS‐SPR displayed more sensitive detection abilities (589 copies/ml) than homemade target‐captured ELISA (4 × 10^3^ copies/ml), SRAT (4 × 10^4^ copies/ml), and comparable to qRT‐PCR (312 copies/ml) when SARS‐CoV‐2 pseudovirus was in PBS solution (Table 1). Regarding the detection of SARS‐CoV‐2 pseudovirus in mimic samples, the PS‐SPR still showed a comparable detection limit to qRT‐PCR (1725 copies/ml and 1250 copies/ml in PS‐SPR and qRT‐PCR, respectively) (Table 1), even though all these diagnostic assays had a reduction in the detection sensitivity. The mimic sample is proposed to contain human respiratory mucus, saliva protease, RNases, and so forth. These components may reduce the SARS‐CoV‐2 virus stabilities via the digestion and degradation of viral protein and viral RNA, subsequently showing the negative impacts in SARS‐CoV‐2 diagnosis and detection. The benefits of the PS‐SPR biosensor include the reduced time consumption and significant increase in the sensitivity of antibody‐based immunodiagnostic assay. PS‐SPR took under 10 min as well as a smaller amount of sample to complete whole diagnosis (Table 1). Current strategies to prevent and control COVID‐19 attack are mainly relied on increase of vaccination rate to enhance the herd immunity as well as early detection of SARS‐CoV‐2 virus to block the transmission and chains of infection. Unfortunately, SARS‐CoV‐2 is still under positive selection by our immune system to continuously generate new variants,^17^, ^18^, ^72^, ^73^ which may lead to decrease of protection efficacy by receiving the COVID‐19 vaccine,^74^, ^75^ enhancement of spike‐ACE2 infection^18^, ^76^ as well as reduction of diagnostic rate by immune‐based assays.^74^, ^77^ Therefore, we believed that the PS‐SPR biosensor integrated with the novel S‐mAb has a high potential to be an efficient diagnostic platform to be applied for SARS‐CoV‐2 virus detection and diagnosis.

**TABLE 1:** Method comparison for SARS‐CoV‐2 detection

Detection assay		qRT‐PCR	Target‐captured ELISA	SRAT	PS‐SPR
Time cost		2.5 h	5 h	15 min	<10 min
Detection target		Nucleic acid	S protein	S protein	S protein
Limit of detection	Recombinant S1 proteins	NA	0.48 ng/ml	NA	11 pg/ml
	Pseudovirus in medium	312	4 × 10^3^	4 × 10^4^	589 (copies/ml)
	Pseudovirus in mimic sample	1250	4 × 10^4^	4 × 10^5^	1725 (copies/ml)

Abbreviations: qRT‐PCR, quantitative reverse transcription polymerase chain reaction; PS‐SPR, phase‐sensitive surface plasmon resonance; SRAT, spike rapid antigen test; SARS‐CoV‐2, severe acute respiratory syndrome coronavirus 2.

## 4 CONCLUSIONS

SARS‐CoV‐2 emerged in late 2019 and is continuously mutating and circulating among the human population. Although the recent SARS‐CoV‐2 variants showed higher adaption abilities to ACE2‐expressed cells and reduction of death rate of COVID‐19 patients, COVID‐19 threat still remains and aggressive development of antiviral drug and diagnostic platform are still necessary. Immuno‐based diagnostic assays have been widely used in clinical virological laboratories. In this study, we generated a novel S‐mAb with abilities to recognize a conserved epitope of most SARS‐CoV‐2 variants and further to be applied for PS‐SPR establishment. Our work reveals that the PS‐SPR biosensor platform showed a sensitive detection of SARS‐CoV‐2 pseudovirus in PBS and mimic sample better than homemade target‐captured ELISA, SRAT, and comparable to qRT‐PCR. Besides, the ability of PS‐SPR to detect SARS‐CoV‐2 in real sample was further demonstrated, and the assay time was less than 10 min. We therefore conclude that the biosensing platform would be suitable for rapid diagnosis of SARS‐CoV‐2 infection.

## AUTHOR CONTRIBUTIONS

**Chih‐Yen Lin:** Methodology (equal); project administration (equal); writing – original draft (equal). **Wen‐Hung Wang:** Methodology (equal); project administration (equal); writing – original draft (equal). **Meng‐Chi Li:** Methodology (equal); project administration (equal); writing – original draft (equal). **Yu‐Ting Lin:** Formal analysis (equal); investigation (equal). **Zih‐Syuan Yang:** Formal analysis (equal); investigation (equal). **Aspiro Nayim Urbina:** Validation (equal); writing – review and editing (supporting). **Wanchai Assavalapsakul:** Formal analysis (supporting); investigation (supporting). **Arunee Thitithanyanont:** Formal analysis (supporting); investigation (supporting). **Kai‐Ren Chen:** Software (equal); validation (equal). **Chien‐Cheng Kuo:** Supervision (equal). **Yu‐Xen Lin:** Software (equal). **Hui‐Hua Hsiao:** Investigation (equal); resources (equal). **Kun‐Der Lin:** Investigation (equal); resources (equal). **Shang‐Yi Lin:** Resources (equal); writing – review and editing (equal). **Yen‐Hsu Chen:** Resources (equal); writing – review and editing (supporting). **Ming‐Lung Yu:** Resources (equal); writing – review and editing (supporting). **Li‐Chen Su:** Conceptualization (equal); funding acquisition (equal); writing – original draft (equal); writing – review and editing (equal). **Sheng‐Fan Wang:** Conceptualization (equal).

## CONFLICT OF INTEREST

The authors declare no potential conflicts of interest.

## Supporting information


**Appendix S1** Supporting Information.

## Data Availability

Data available in article supplementary material.
