# Supplementary material for: Boosting the detection performance of severe acute respiratory syndrome coronavirus 2 test through a sensitive optical biosensor with new superior antibody
Source: Bioeng Transl Med. 2022 Sep 16;8(5):e10410. doi: 10.1002/btm2.10410 (PMC9538096; doi:10.1002/btm2.10410)
Supplement: Supplementary file 1 — Appendix S1 Supporting Information. [file BTM2-8-e10410-s001.docx]

**Boosting the detection performance of SARS-CoV-2 test through a sensitive optical biosensor with new superior antibody**

Chih-Yen Lin^1,2,#^, Wen-Hung Wang^2,3,4,#^, Meng-Chi Li^5,6,#^, Yu-Ting Lin^1,2^, Zih-Syuan Yang^1,2^, Aspiro Nayim Urbina^2^, Wanchai Assavalapsakul^7^, Arunee Thitithanyanont^8^, Kai-Ren Chen^9^, Chien-Cheng Kuo^5,9^, Yu-Xen Lin^10^, Hui-Hua Hsiao^11^, Kun-Der Lin^12^, Shang-Yi Lin^4,13^, Yen-Hsu Chen^2,3,4^, Ming-Lung Yu^3,14^, Li-Chen Su^15,16,*^, Sheng-Fan Wang^1, 2, 17*^

^1^ Department of Medical Laboratory Science and Biotechnology, Kaohsiung Medical University, Kaohsiung 80708, Taiwan.

^2^ Center for Tropical Medicine and Infectious Disease Research, Kaohsiung Medical University, Kaohsiung 80708, Taiwan.

^3.^School of Medicine, College of Medicine, National Sun Yat-Sen University, Kaohsiung 804201, Taiwan

^4^ Division of Infection Disease, Department of Internal Medicine, Kaohsiung Medical University Hospital, Kaohsiung 80708, Taiwan.

^5^ Thin Film Technology Center, National Central University, Taoyuan 32001, Taiwan.

^6^ Optical Sciences Center, National Central University, Taoyuan 32001, Taiwan.

^7^ Department of Microbiology, Faculty of Science, Chulalongkorn University, Bangkok 10330, Thailand.

^8^ Department of Microbiology, Faculty of Science, Mahidol University, Bangkok 10400, Thailand.

^9^ Department of Optics and Photonics, National Central University, Taoyuan 32001, Taiwan.

^10^ TeraOptics Corporation, Taoyuan 32472, Taiwan.

^11.^Division of Hematology and Oncology, Department of Internal Medicine, Kaohsiung Medical University Hospital, Kaohsiung 80708, Taiwan.

^12.^Division of Endocrinology and Metabolism, Kaohsiung Medical University Hospital, Kaohsiung Medical University,

Kaohsiung 80708, Taiwan.

^13^ Department of Laboratory Medicine, Kaohsiung Medical University Hospital, Kaohsiung 80708, Taiwan.

^14^ Hepatobiliary Section, Department of Internal Medicine, and Hepatitis Center, Kaohsiung Medical University Hospital, Kaohsiung 80708, Taiwan.

^15^ General Education Center, Ming Chi University of Technology, New Taipei City 24301, Taiwan.

^16^ Organic Electronics Research Center, Ming Chi University of Technology, New Taipei City 24301, Taiwan.

^17^ Department of Medical Research, Kaohsiung Medical University Hospital, Kaohsiung 80708, Taiwan.

^#^ These authors equally contributed to this work.

***Corresponding Author:**

Dr. Li-Chen Su (e-mail: [sulichen@o365.mcut.edu.tw](mailto:sulichen@o365.mcut.edu.tw)), General Education Center, Ming Chi University of Technology, New Taipei City 243303, Taiwan. Tel: +886-2-29089899#4533; Fax: +886-2-2904-1914.

Dr. Sheng-Fan Wang (e-mail: wasf1234@kmu.edu.tw), Department of Medical Laboratory Science and Biotechnology, Kaohsiung Medical University, Kaohsiung 80708, Taiwan. Tel: +886-7-3121101#2558; Fax: +886-7-322-2783.

Supplementary data

METHODS

**SASR-CoV-2 spike-ACE2 protein-protein interaction assay**

The SASR-CoV-2 spike-ACE2 protein-protein interaction assay was described elsewhere.^1^ Briefly, 96-well plates (Nunc, Roskilde, Demark) were coated with 0.1 µg recombinant ACE2-Fc protein (SinoBiological, Cat. no. 10108-H05H) in 100 μL carbonate buffer (73 mM sodium bicarbonate and 30 mM sodium carbonate) and incubated at 4°C overnight. The plates were washed three times with PBST (PBS pH 7.4, 0.05% Tween-20) and blocked with PBST that contained 5% bovine serum albumin (BSA) at 37°C for 1hr. After washing three times with PBST, 100μL Biotinylated-SARS-CoV-2 spike S1 protein (SinoBiological, Cat. No. 40591-V08H) (1μg/mL) was added into each well and incubated at 37$℃$ for 2hrs. Notably, the biotinylated-SARS-CoV-2 spike S1 protein was pre-incubated with anti-sera from COVID-19 convalescent patients or vaccinees prior to being added to each well. After washing three five with PBST buffer, 100 μL streptavidin-HRP (Abcam, cat. no. ab7403) was added into each well. After washing five times with PBST, color development was performed by the addition of 100 μL of freshly prepared TMB (solutiontetramethyl benzidine) solution (ThermoFisher); the absorbance at 450 nm was read with an ELISA reader (Tecan, Switzerland).

**SARS-CoV-2 pseudovirus generation**

SARS-CoV-2 pseudovirus was generated according to the protocol published previously.^2^ Briefly, the pNL4-3.Luc.R-E- (NIH AIDS Reagent program, Cat.No. ARP-3418) and pSARS-CoV-2 S (SinoBiological Cat.No.VG40589-UT) (the ratio 2:1) were co-transfected to 293T cells to generate recombinant pseudotyped HIV particles containing SARS-CoV-2 spike envelope glycoproteins protein (Genbank Accession # YP_009724390.1) using Effectene transfection reagent (Qiagen, Cat. No.301425). The supernatant containing SARS-CoV-2 pseudoviruses were harvested 48hr after transfection and filtered through a 0.45 mm filter. These pseudovirions also contain the luciferase (Luc) gene, therefore, the spike-mediated cell entry can be conveniently determined via luciferase activity. The generated SARS-CoV-2 pseudovirus titers were determined using p24 ELISA or qRT-PCR. The SARS-CoV-2 pseudovirus can be used to measure the activity of neutralizing antibody against SARS-CoV-2 in a Biosafety Level 2 facility.

**Infectivity assay using SARS-CoV-2 pseudovirus**

HEK293 expressed human ACE2 cells (293T-ACE2) (1x10^4^ cells/well) were seeded into 96-well plates and infected with 50 μL of the pseudotyped viruses supplemented with polybrene (5 mg/mL). Note that some of SARS-CoV-2 pseudovirus were co-incubated with anti-sera from COVID-19 vaccinees or recovered patients. After incubation for 12 h, the pseudovirus-containing supernatant was removed and replaced with fresh DMEM containing 10% FBS. After 48-72 h post-infection, the 293T-ACE2 cells were collected and subjected to measurement of the luciferase activity or immune-fluorescent staining with anti-p24 antibody (Invitrogen, Cat.No.PA5-81775). Relative luminescence units (RLU) of luciferase activity was detected using the Luciferase Assay Kit (Promega). All experiments were performed at least three times and expressed as means ± standard deviations (SDs).

**Reference**

1. Tan CW, Chia WN, Qin X, et al. A SARS-CoV-2 surrogate virus neutralization test based on antibody-mediated blockage of ACE2-spike protein-protein interaction. 2020; **38**(9): 1073-1078.

2. Hu J, Gao Q, He C, et al. Development of cell-based pseudovirus entry assay to identify potential viral entry inhibitors and neutralizing antibodies against SARS-CoV-2. 2020; **7**(4): 551-557.

Results

**S-Figure 1. The molecular weight and functional validation of SASR-CoV-2 spike S1 protein.** The SASR-CoV-2 spike S1 recombinant protein expressed via HEK293T cells was subjected to (A) Coomassie staining, (B) immunoblotting analysis with COVID-19 convalescent anti-sera and (C) Spike-ACE2 protein-protein ELISA based assay. The representative data are shown. Results are presented as the means + standard deviations (SDs) of three independent biological replicates.

**S-Figure 2. The comparison of binding capabilities between the newly-developed S-mAb and commercial S-mAbs using ELISA assay with the spike proteins from different SARS-CoV-2 variants.** Our generated S-mAb was used to compare the binding capabilities toward spike proteins with two commercial S-mAb (invirogen-GT236 & Abcam-8B12-C2). (A)-(F) indicate different detection spike variants.

**S-Figure 3. The comparison of binding capabilities between the newly-developed S-mAb and commercial S-mAbs using immunoblotting assay with the spike proteins from different SARS-CoV-2 variants.**

**S-Figure 4. Specificity analysis of selected S-mAbs towards SARS-CoV-1 and SARS-CoV-2 detection.** Our generated S-mAbs could recognize 4 different epitopes beside spike RBD domain. We selected the representative mAbs 8-8G, 10-6G, 10-9A and 10-11G which respectively recognized these 4 epitopes and subjected them to (A) ELISA and (B) Immunoblotting.

**S-Figure 5. The functional validation of** **SASR-CoV-2 pseudovirus.** SARS-CoV-2 spike pseudotyped lentivirus containing luciferase (Luc) gene was generated and subjected to infectivity assay using HEK293T human ACE2 expressing cells (239T-ACE2). The SASR-CoV-2 pseudovirus were co-treated with anti-sera from healthy individuals or COVID-19 recovered patients prior to be incubated with 293T-ACE2 cells. (A) The luciferase activity of infectious 293T-ACE2 cells was measured. (B) The infectious 293T-ACE2 cells were subjected to immunofluorescent assay with p24 mAb. The RLU indicates relative luminescence units. The data are presented as the means + standard deviations (SDs) of three independent biological replicates.

(“**”, p<0.01).

**S-Figure 6. Detection sensitivity comparison between homemade target-captured ELISA and commercial SARS-CoV-2 Spike ELISA kit.** The standard curve establishment for S1 protein detection by (A) homemade target-captured ELISA and (C) commercial SARS-CoV-2 Spike ELISA kit. The serial diluted SARS-CoV-2 pseudoviruses were subjected for detection by (B) homemade target-captured ELISA and (D) commercial SARS-CoV-2 Spike ELISA kit.

**S-Figure 7. Validation of detective sensitivity of SASR-CoV-2 pseudovirus in PBS or** **mimic samples using PS-SPR.** The dynamic SPR signals for various concentrations of SARS-CoV-2 pseudovirus in (A) PBS and (B) mimic samples ranging from 10 copies/mL to 4.0×10^5^ copies/mL.

# S-Table 1. Analysis of binding affinity of generated monoclonal antibody against spike protein using surface plasmon resonance assay

| **Anti-S Antibody**  **No.** | **k_a_ (1/Ms)** | **k_d_ (1/s)** | **K_D_ (M)** |
| --- | --- | --- | --- |
| PAb | 4.740E+04 | 3.400E-04 | 7.490E-09 |
| 10-11G | 1.370E+04 | 4.337E-03 | 3.170E-07 |
| 11-3F | 1.278E+04 | 3.699E-03 | 3.469E-07 |
| 11-8H | 5.729E+03 | 4.251E-03 | 7.747E-07 |
| 10-6G | 4.556E+03 | 3.339E-03 | 8.682E-07 |
| 10-11C | 6.540E+03 | 5.550E-03 | 1.080E-06 |
| 11-12H | 2.605E+03 | 4.153E-03 | 1.970E-06 |
| 8-8G | 1.869E+03 | 3.499E-03 | 1.974E-06 |
| 10-9A | 2.403E+03 | 3.187E-03 | 2.111E-06 |
| 11-9D | 1.327E+03 | 3.445E-03 | 2.744E-06 |
| 11-12H | 9.509E+02 | 3.317E-03 | 3.490E-06 |

**S-Table 2.** Detection of COVID-19 in the clinical samples.

| Ct value | negative | 37 | 36 | 33 | 29 | 28 | 25 | 24 | 21 | 17 |
| --- | --- | --- | --- | --- | --- | --- | --- | --- | --- | --- |
| Result | - | - | - | - | + | + | + | + | + | + |
